# 3D modeling of cancer stem cell niche

**DOI:** 10.18632/oncotarget.19847

**Published:** 2017-08-03

**Authors:** Jun He, Li Xiong, Qinglong Li, Liangwu Lin, Xiongying Miao, Shichao Yan, Zhangyong Hong, Leping Yang, Yu Wen, Xiyun Deng

**Affiliations:** ^1^ Department of General Surgery, Second Xiangya Hospital, Central South University, Changsha, Hunan, China; ^2^ State Key Laboratory of Powder Metallurgy, Central South University, Changsha, Hunan, China; ^3^ Department of Pathology, Hunan Normal University Medical College, Changsha, Hunan, China; ^4^ State Key Laboratory of Medicinal Chemical Biology, College of Life Sciences, Nankai University, Tianjin, China

**Keywords:** cancer stem cells, niche, 3D models

## Abstract

Cancer stem cells reside in a distinct microenvironment called niche. The reciprocal interactions between cancer stem cells and niche contribute to the maintenance and enrichment of cancer stem cells. In order to simulate the interactions between cancer stem cells and niche, three-dimensional models have been developed. These *in vitro* culture systems recapitulate the spatial dimension, cellular heterogeneity, and the molecular networks of the tumor microenvironment and show great promise in elucidating the pathophysiology of cancer stem cells and designing more clinically relavant treatment modalites.

## INTRODUCTION

Cancer stem cells (CSCs) or tumor-initiating cells (TICs) are a special subpopulation in cancer tissues which perform self-renewal to maintain the pool of progenitor cells and differentiate to regenerate tumor cells in malignant tissues [1–3]. CSCs have long-term clone-propagating capacity and can generate progeny with self-limiting ability of proliferation [4, 5]. Classical tumor model claims that the origin of cancer cell is randomly selected if equipped with certain gene mutations which are remarkably influenced by tumor microenvironment [6, 7]. When non-stem cells are modified with stem-associated genes or gene products, they can display the traits of stemness [8–11]. Recent findings suggest that phenotypic plasticity between differentiated cells and stem cells engages in the generation of CSCs and the bulk tumor cells. An excellent review concludes that tumor microenvironment also exerts significant impact on the cellular plasticity between the CSCs and the non-CSCs [7].

Heterogeneity is inevitable to address the characteristics and hallmarks of tumors [12]. The intrinsic heterogeneity is attributed to heterogeneous entities of tumor tissues, which consist of CSCs, bulk tumor cells, stromal cells, and endothelial cells. Additionally, a designated cell type such as CSC might also have significant heterogeneity. Considering the cancer cell plasticity, i.e., bulk cells may be able to re-acquire stem cell traits, some studies propose CSCs are more a state of tumor cells rather than a real existing entity [13]. In normal tissues, stem cells with multiple levels of maturation exhibit diverse morphologies, molecular characteristics, and notably distinct functions, which contribute to establish and modulate the tissue homeostasis. A bold speculation is that the primary CSCs would generate differentiated progenitors which transform to terminally differentiated tumor cells.

Tumor microenvironment or niche is a major factor that extrinsically influences the tumor heterogeneity. Niche is comprised of stromal cells, immune cells, endothelial cells, and cancer cells *per se*, as well as connective tissue components, growth factors, and cytokines [14]. Niche plays an essential role in CSC maintenance/enrichment, preservation of the phenotypic plasticity, immune-surveillance, differentiation/dedifferentiation, angiogenesis activation and invasion/metastasis [15–17].

In order to investigate the interactions between niche and CSCs and to better reflect heterogeneity, three-dimensional (3D) culture systems are developed to recapitulate the spatial dimension, cellular heterogeneity, and the molecular networks of the tumor microenvironment. As robust progresses are made in tissue engineering, tumor models, culture technologies and surveillance methods *in vitro*, sophisticated 3D models are proposed with the potential to further understand the CSCs in a more appropriate condition resembling the *in vivo* microenvironment. This review focuses on the recent progress in our understanding of the interactions between CSCs and niche, with a special emphasis on the various 3D models and their respective applications for context-dependent pathophysiological behaviors.

### The influence of niche on CSCs

#### Effect of niche on the maintenance and enrichment of CSCs

**Figure 1:**
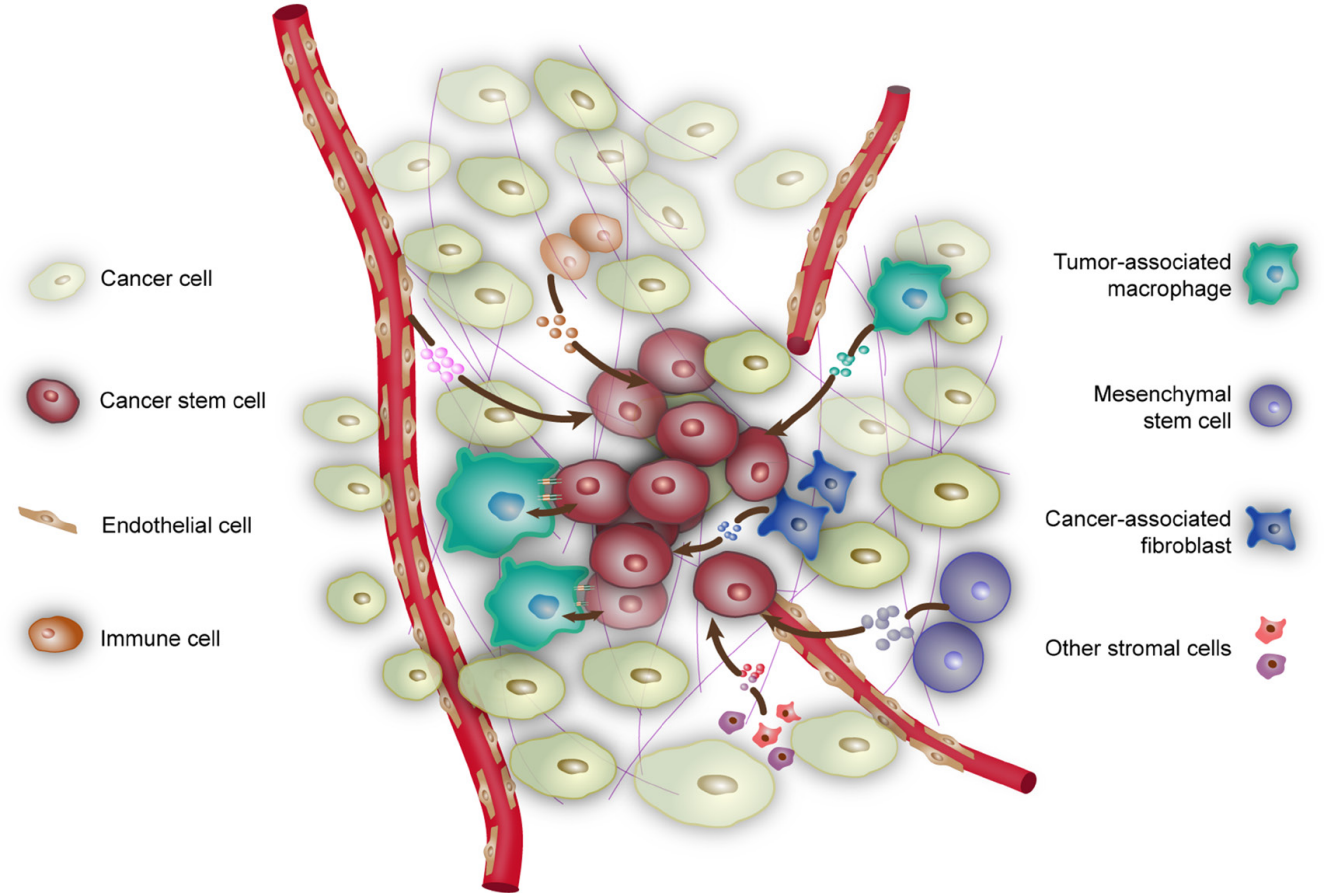
Niche contributes to the maintenance of CSCs Niche is composed of cancer cells, various non-cancer cells, as well as physical and biochemical factors that maintain CSCs. Tumor-associated macrophages exert influence on CSCs by direct contact or through soluble factors such as EGF and ISG15. Mesemchymal stem cells secrete cytokines such as PGE_2_, IL-6, IL-8, and Gro-α. Endothelial cells and vessels provide nutrition and oxygen to support CSCs. In turn, CSCs produce VEGF and SDF1 to stimulate angiogenesis. Cancer-associated fibroblasts release a variety of growth factors, chemokines, and components of the ECM into niche, such as AnxA1, IGF-II, HGF, LIF, and SDF1. In addition, hypoxia can also contribute to the maintainence and formation of CSCs.

CSCs reside in a niche which not only provides the physical support for CSCs but also fundamentally influences the functional status of CSCs. Tumor can locally and metastatically colonize at the proper sites, where CSCs play an essential role in these processes. The bulk tumor preferentially exists in a relatively dormant state where the existence of CSCs is responsible for the resuscitation and restoration of tumors. Various niche factors influence the proliferation and self-renewal of CSCs. It is conceivable that the signaling pathways involved in cell cycle, growth factor secretion, and stemness properties would be activated that elicit stimulation on CSCs in niche. In turn, tumor cells may contribute to the formation and maintenance of niche. A schematic of the components of niche and their interactions with CSCs is presented in Figure 1.

The stemness is often defined by high expression of putative stemness markers, great capacity of tumorsphere formation, and significant tumorigenicity *in vivo.* These features can be explained by several attributes. First, culture conditions might exert rather heterogeneous influences on cell proliferation and apoptosis in diverse subpopulations derived from the same tumors. CSCs which are generally more resistant to numerous pernicious cues such as hypoxia and nutrition depletion would proliferate with much prevailing rate over the more susceptible non-stem cells. Second, it is reasoned that the non-stem cells identified by current methods might conceal some real CSCs, in light of the fact that different stem markers indicative of CSCs are relatively exclusive and inconsistent and the sorted subpopulations show insufficient overlaps with each other. Third, mature and terminal cells can be reprogrammed and dedifferentiate into CSCs. The prevailing proliferation rate of CSCs is the major determinant to organize heterogeneous tumors in primary or metastatic sites. Concomitantly, stronger resistance of the CSCs to niche stress, including hypoxia, cytotoxic T lymphocytes, chemotherapy, and radiotherapy, provides competitive advantages compared to the bulk tumor cells. To elucidate the mechanisms of cancer heterogeneity, the process of dedifferentiation or reprogramming deserves more attentions, in virtue of the overlapping signaling pathways such as Wnt and TGF-β1 in the maintenance of stemness and mediating dedifferentiation [18, 19] .

#### Effect of niche on the metastasis of CSCs

**Figure 2:**
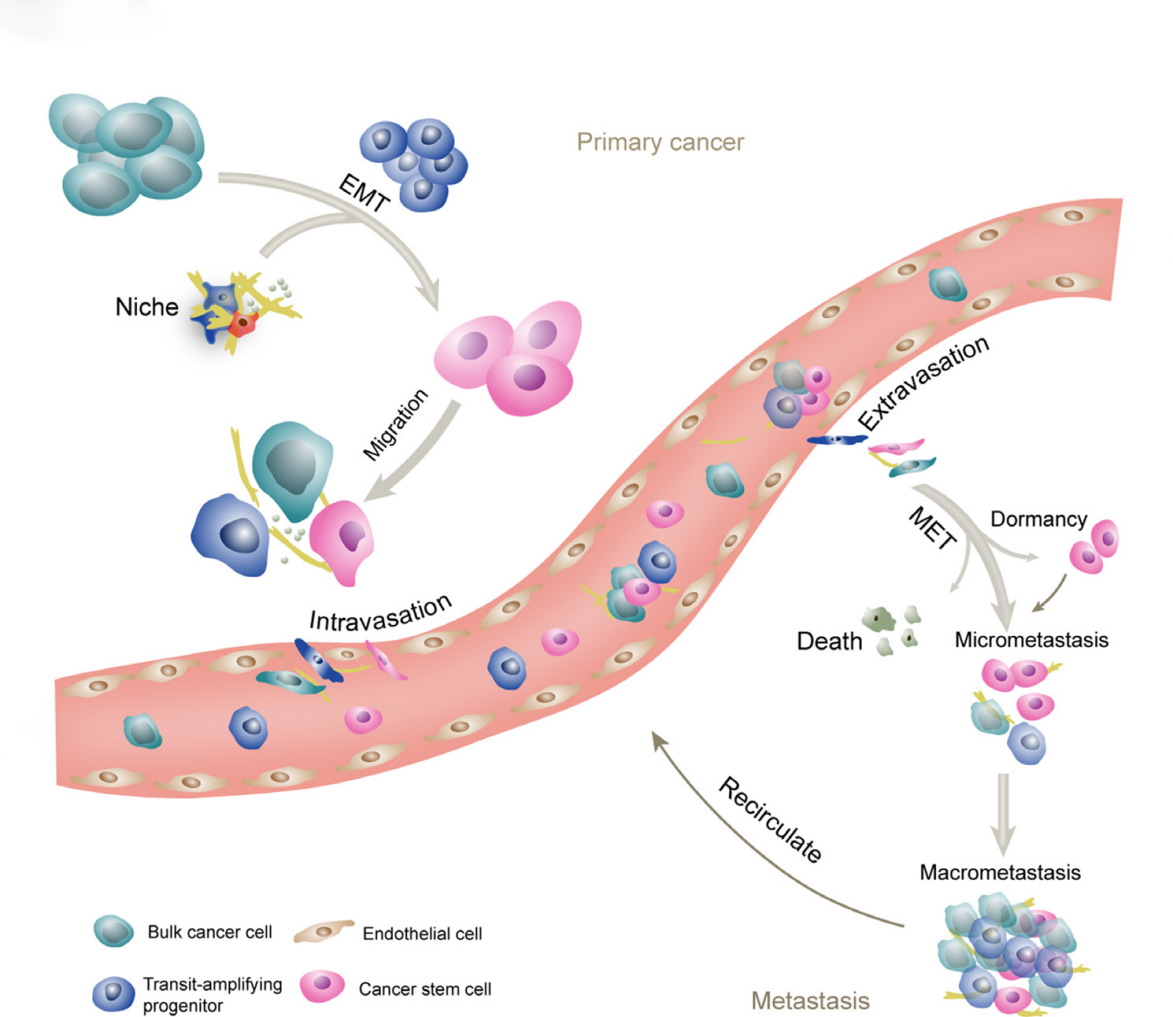
The schematic of CSCs and metastasis Metastatic cascade involves invasion and intravasation from the primary tumor, circulation and transformation in the vessel systems, selective extravasation in certain organs, survival and settlement into the foreign niches, reactivation from cell cycle arrest, and re-building of an overt tumor mass. CSCs are regarded as the initiating cells in the primary tumor and at the metastatic sites. The transit-amplifying progenitors are derived from CSCs and committed to generate differentiated cancer cells. The EMT program leads to generation of the CSC phenotype, while the reverse process will facilitate the establishment of metastatic tumors. Collective invasion and collective circulation are important ways for the cancer cells to enhance the efficiency of metastasis. Frequently, after extravasation, the cancer cells enter a dormant state that can last for decades and exhibit strong resistance to current therapies. In appropriate situation, the micrometastasis will progress into the macrometastasis.

The broad designation of stemness should encompass that CSCs are translated from primary sites through vessels or lymphatics to distant tissues, and regenerate secondary tumors. Metastatic cascade involves invasion and intravasation from the primary tumor, circulation and transformation in the vessel systems, selective extravasation in certain organs, survival and settlement in the distant site, and reactivation from cell cycle arrest, and re-building an overt tumor mass from micrometastasis. These processes associated with CSCs are shown in Figure 2. To elucidate the relationship between CSCs and metastasis, consecutive tracking and monitoring should be conducted. However, currently, only intermittent preclinical evidence is available to suggest the role of CSCs in disseminating tumors.

Multiple lines of evidence suggest that the enrichment of CSCs is positively associated with late-stage cancers and can serve as a poor prognosis predictor in cancer patients [3, 20–25]. In breast cancer, for example, the expression of the CSC marker ALDH1 is closely correlated with the development of distant metastasis and decreased survival in patients with inflammatory breast cancer [26]. To interrogate the homogeneity of primary and metastatic CSCs, large-scale genome sequencing studies suggest that the mutations which are vital for metastasis show a predominant similarity between metastatic and primary CSCs [27]. CSCs unequivocally exist in vessels or lymphatics and can generate metastasis in multiple organs, including bone, liver, lung, etc [28]. In the hostile circulatory system, CSCs might survive through multicellular assemblies and be transformed in a dormant state [29]. On the other hand, perivascular cells secret factors such as VEGF to support CSC survival, anoikis resistance, invasiveness and tumor vasculogenesis in the CSC-microvascular niche and the invasive tumor edge [30].

CSCs shed from the primary sites circulate and locate in metastatic regions. Under great stress of metastatic elimination, only a tiny fraction of disseminated cancer cells can survive and initiate metastatic outgrowth. The term “metastatic niche” is used to designate the specific locations, stromal cells, diffusible signals, and extracellular matrix (ECM) components that could function on survival, self-renewal, and tumorigenesis of metastatic stem cells [31–34]. The microenvironmental components are delineated in the next section.

Tumor cells reside in an acidic niche resulting from a marked fluctuation of glucose and lactate [35]. The vasculatures of tumors are chaotic and incomplete with transient or persistent oxygen deficiency. Thus, tumors preferably conduct an anaerobic glycolysis resulting in a greater amount of lactic acid and a lower tissue pH [36]. Tumor acidity endows cancer cells with several advantages for progression and metastasis, including remodeling of ECM, activating proteases such as cathepsins and gelatinases, and stimulating angiogenesis and lymphangiogenesis through enhanced release of vascular endothelial growth factor (VEGF) A and C [35, 37]. Furthermore, acidic niche can enhance the EMT process, as acidity renders the cells a more spindle-like shape, and enhances the expression of EMT-associated markers [38]. The effect of acidity in inducing mesenchymal phenotype switch is largely mediated through the NF-κB signaling pathway [39].

#### Effect of niche on the EMT/MET program of CSCs

Epithelial-to-mesenchymal transition (EMT) facilitates invasion and intravasation of neoplastic cells [40, 41]. This allows conversion of polarized epithelial cells to mesenchymal-like cells with characteristics of spindle shape and more motility [42]. Intriguingly, substantial evidence assists to establish the close link between EMT and CSCs. EMT program is responsible for the generation of CSCs and promoting invasive and metastatic phenotype in various cancer types [43, 44]. Breast cancer cells compose a high proportion of putative CSCs with basal/mesenchymal phenotype [45]. The induction of EMT is modulated by an intricate network of multiple signaling pathways and some of them demonstrate close overlap with signals important for the maintenance and enrichment of stemness, including TGF-β, Wnt, Notch, NF-κB, and ERK/MAPK pathways [46]. In addition, the microRNAs, miR200 and miR21 are important components of the cellular signaling circuitry which modulates the EMT program and connects with CSC signatures [47]. Enforced activation of such signaling pathways in cancers would lead to the acquisition of the EMT phenotype, as well as enhanced ability of self-renewal, spheroid formation, and tumor generation. Various microenvironmental factors are convincingly capable of inducing EMT and CSCs. Albeit scarce of systematic identifications, some scattered documents are presented. For example, the tumor microenvironmental factor, FOXC2, functions as a central mediator of EMT and is independent of the initiating signals [44].

The circulating tumor cells (CTCs) or CSCs detected in the circulation show low activity and dormancy, which contributes to chemo-resistance and survival advantage over active cells. Nevertheless, the mesenchymal cells produced via complete EMT exhibit insufficient ability to generate macrometastasis. Thus, mesenchymal-to-epithelial transition (MET), the reverse program of EMT, is performed to reacquire the epithelial phenotype. The relationship between CSCs and MET is implicated in several reports. In breast cancer, the number of sorted CTCs expressing putative stem markers and MET markers is a better indicator for patient overall survival and metastatic burden than bulk CTCs [28]. It is proposed that CSCs may exist in either EMT or MET states and the inter-conversion of them is regulated by the microenvironment. For example, TGF-β generated in the tumor niche induces the program of EMT, whereas inhibition of TGF-β signaling and stimulation of BMPs will induce MET. CSCs signal transduction pathways including Wnt and NF-κB can induce EMT, while HER2 induce the program of MET [48, 49]. Actually, the correlation between CSCs and MET is much speculative and short of corroborative experimental evidence because of the scarcity of MET population *per se* and the lack of faithful isolation techniques.

#### Preservation of the dynamic equilibrium of diverse subpopulations in cancers

In the classical stem cell model, an accumulation of unique mutations and low allelic frequencies will occur in non-stem cells as a consequence of high proliferation and irreversible conversions from stem cells to differentiated cells. New findings have revealed a more dynamic model in CSCs, i.e., there exists a reversible inter-conversion in the tumor between the CSCs and the non-CSCs [13, 50–53]. Thus, it is reasoned that the mutations and their allelic frequencies would not differ between CSCs and non-CSCs, while the major differences should reside in the epigenetic profile. Indeed, a CSC might be generated from a differentiated cancer cell via epigenetic modulation [54]. Exome sequencing revealed significant overlap of shared mutations between the CSCs and bulk primary tumor cells and the frequency of each mutation is also similar. In addition, an array of genetic markers commonly altered in breast cancer indicate a very similar genetic diversity between putative CSCs and non-CSCs. Thus, the variation of gene profile in CSCs and non-CSCs may not be the major player in mediating the balance of different cell types, which might be predominantly determined by the epigenetic factors [55].

The inter-conversion between differentiated cancer cells and CSCs suggests a dynamic equilibrium of diverse subpopulations *in vitro* and *in vivo*. Under fixed culture conditions, breast cancer cell lines display stable proportions of various cell types including stem-like, basal, and luminal cells [55]. The isolated pure subpopulations with those cell types consistently exhibit the similar phenotype equilibrium after appropriate propagation *in vitro*. In melanoma, either the offspring of differentiated cells or putative CSCs could re-establish a hierarchy containing various subtypes under long-term culture with conventional medium [56]. Strikingly, a Markov model of cell-state dynamics was proposed which assumes that the inter-convertion rate depends only on cells’ current state and remains constant under fixed microenvironmental conditions [57]. The notion is that intercellular signals clearly influence cell-state decisions, but they are not necessarily required for the phenotypic stability. Thus, the niche where CSCs reside in might be the major determinant of the dynamic equilibrium of diverse subpopulations in cancers.

### The niche components that contribute to the stemness of CSCs

#### Tumor-associated macrophages

Tumor-associated macrophages (TAMs) are important tumor-infiltrating inflammatory cells and the mechanisms by which these cells modulate CSCs have been exploited intensively [58, 59]. EGFR/STAT3 signaling pathway and the downstream transcription factor Sox2 engage in modulating the proliferation and enrichment of CSCs by TAMs [60, 61]. NF-κB, when activated, enters the nucleus and, in collaboration with the master EMT regulator Twist, to enhance the production of cytokines, including IL-6, IL-8, and GM-CSF [62]. TAMs employ another vital pathway to maintain CSCs, namely the ISG15 signaling pathway [63]. Recombinant ISG15 pronouncedly elevates the self-renewal and sphere-forming capacity in pancreatic ductal adenocarcinoma. Evidence supports that the AKT and probably ERK1/2 signaling pathways play a predominant role in ISG15-mediated downstream effects in pancreatic ductal adenocarcinoma CSCs [64, 65]. Additionally, Leucine leucine-37 (LL-37) could be incorporated into CSCs and significantly potentiates the CSC stemness [66]. Indeed, the reciprocal relationship between TAMs and CSCs has been demonstrated experimentally [67].

#### Endothelial cells

Tumor vasculature plays an essential role in tumor development and progression [68]. Tumor cells often reside in a relative hypoxic niche, and show sufficient capacity of death-resistance [69]. Corroborative evidence supports the close proximal localization of CSCs and vessels [70–73]. The functional interdependency between CSCs and endothelial cells has also been documented in multiple studies [70, 71]. CSCs participate in the process of angiogenesis through stimulating proliferation and/or differentiation of endothelial cells to generate a vessel-rich niche [74, 75]. These effects are predominantly mediated by angio-reactive factors released from CSCs [76, 77]. In addition, CSCs can promote homing and recruitment of endothelial progenitor cells, and probably also influence other bone marrow-derived cells, such as hemangiocytes. On the other hand, endothelial cell-derived factors could contribute to the survival, proliferation, and self-renewal of CSCs [71].

#### Cancer-associated fibroblasts

Cancer-associated fibroblasts (CAFs) are activated fibroblasts that share similarities with normal fibroblasts and are stimulated by inflammatory conditions related to cancer development [78]. They constitute a significant component of the stroma that surround cancer cells and play an important role, not only in mechanical support, but in proliferation, survival, angiogenesis, metastasis, and immunogenicity in cancer tissues [79, 80]. Recent emphasis on CSCs indicates the aforementioned behaviros could be at least partially attributed to CSCs interacting with CAFs [81–83]. The stemness properties can be enhanced by conditioned medium from CAFs, suggesting the presence of paracrine-acting secreted molecules to prevent their potential of differentiation [81]. Wnt activity can be enhanced through Hepatocyte growth factor (HGF) binding to its receptor HGFR (or called c-Met), followed by translocation of β-catenin to the nucleus and transcription of stemness-related proteins such as c-Myc [18]. Another important factor from CAFs is AnxA1, which activates the downstream signaling via MAPK extracellular signal-regulated kinase (ERK)-1/2 [81]. Furthermore, the IGF-II/IGF1R signaling plays an important role in the CAF-CSC interaction, in that IGF-II activates IGF1R, followed by phosphorylation of Akt and expression of Nanog [84]. TGF-β receptor ligands engage in the maintenance of CSC stemness mediated by Smad2 [19, 85]. Additionally, the C-C chemokine CCL2 released from CAFs activates p38 signaling and enhances the expression of Notch1 ligand which is mediated by transcrition factors E12 and E47, resulting in enhanced conversion of non-CSCs to CSCs [86, 87].

#### Mesenchymal stem cells

Mesenchymal stem cells (MSCs) are a heterogeneous subset of stromal stem cells which can be isolated from many adult tissues, and can be recruited to tumor sites [88, 89]. The interrelation between MSCs and cancer cells is full of contradictions, which is either indicative of promoting or inhibiting tumor progression within the same cancer model [89]. From a broad insight, the effect of MSCs can tremendously vary depending on numerous factors, including the origin, cancer type, research model, and the relative number of MSCs and cancer cells. MSCs can provide an advantageous microenvironment for the restoration and maintenance of CSCs [90]. The physical relationship between MSCs and CSCs is crucial to elicit these effects. Furthermore, the interaction between MSCs and CSCs is probably based on a complex cytokine network, involving CXCL7, IL-6, IL-8, and CXCL5 [91].

#### Hypoxia

**Figure 3:**
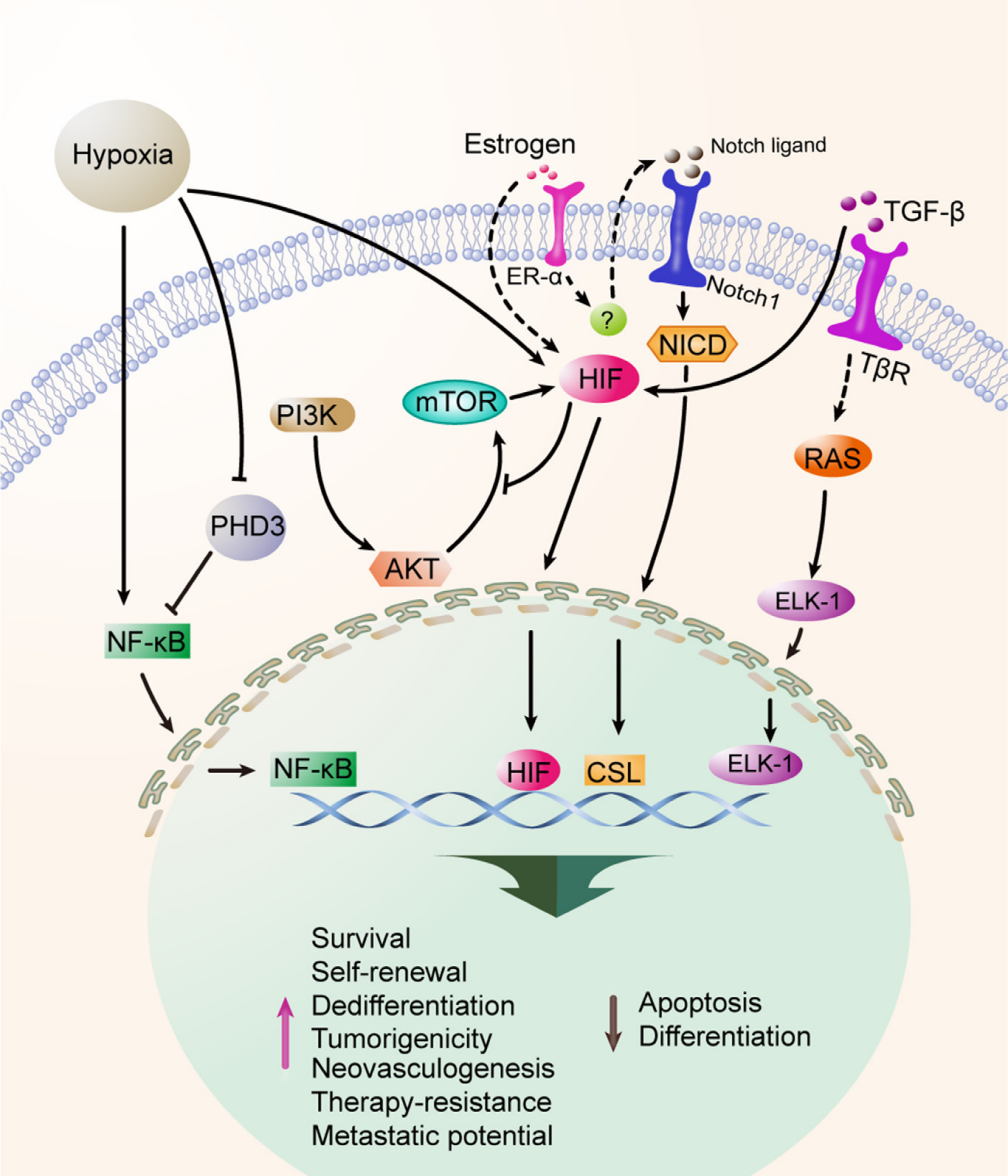
Hypoxia maintians and enriches CSCs Hypoxia is a common characteristic of solid malignancies, which is involved in the processes of self-renewal, stemness maintenance, and EMT of CSCs. Hypoxia-inducible factors (HIFs) are the main effecters on CSC biology under hypoxia and are associated with the Akt, mTOR, Notch, TGF-β and ER-α signaling pathways. ER-α also participates in the response to hypoxia of CSCs. Additionally, the HIF-independent pathway is also implicated that downregulation of prolyl hydroxylase 3 (PHD3) can expand the CSC population.

Hypoxia is a common characteristic of all solid cancers, and indicates a hallmark of disease progression and poor prognosis [92, 93]. Recent evidence suggests that hypoxia is closely associated with EMT [94] and influences the self-renewal, induction and maintenance of the dedifferentiation state, and the enrichment for CSCs [95–97]. Stem-associated genes, including DLK1 and Oct4, are significantly elevated in tumor cells under hypoxic condition, suggesting that hypoxia promotes the stemness properties of CSCs [9, 98–100]. Hypoxia-induced effects are primarily modulated by hypoxia-inducible factors (HIFs) which are highly conserved transcription factors [101–103]. Several signaling pathways play critical roles in the survival and enrichment of CSCs under hypoxia, as illustrated in Figure 3.

Notch signaling is known to be activated under hypoxia in aggressive tumors [104]. The expression of Notch intracellular domain is significantly up-regulated at the invasive front in breast cancer. Hypoxia profoundly up-regulates the expression of Notch ligand Jagged-2 and HIF1α is involved in this process. Furthermore, bone is a major metastatic site for many cancer types where hypoxic pre-metastatic niche is found. Strikingly, stromal cells in bone express high level of Jagged-2 and Notch signaling is notably up-regulated under hypoxic condition [105]. Notch signaling promotes CSC survival under hypoxia and knockdown of Jagged-2 leads to significantly attenuated cell survival in GBM. Because Akt signaling is dramatically decreased after inhibition of Notch intracellular domains, the cross-talk between Notch and Akt signaling is implicated, which contributes to the survival of CSCs under hypoxic condition [105]. On the other hand, hypoxia exerts passive influences on mTOR signaling, which integrates growth factor signaling, cell metabolism, and diverse cellular stressors, modulating the adaptation of proliferation, apoptosis, autophagy, and protein translation. Thus, the signaling axis P13K/Akt/mTOR is established and adopted for the survival and stemness maintenance of CSCs under hypoxia [106]. ER-α participates in the response to hypoxia, as ER-α-positive primary samples and cell lines exhibit a significantly higher mammosphere-forming capacity in contrast to ER-α-negative cells under hypoxia. Notch1 is further verified as a downstream paracrine mediator of ER-α and HIF1α-ER-α-Notch1 is established to enhance stemness found in ER-α-positive cells [107]. TGF-β signaling pathway is responsible for tumor cell dedifferentiation induced by hypoxia [19]. These replenish the understanding of HIF1α-mediated signaling axis in functioning as an essential modulator of the maintenance and enrichment of CSCs under hypoxia. In addition to HIF-dependent effects, HIF-independent hypoxic effects are also documented in some studies [108].

### Floating sphere culture of CSCs

Tumorspheres are floating spherical CSC models and widely applied to CSC study. Tumorspheres can originate from immortal cell lines or fresh patient-derived samples. Technically, to obtain this reservoir for CSCs, cancer cells are cultured in serum-free medium and supplemented with various factors, including basic fibroblast growth factor (bFGF) and epidermal growth factor (EGF), hydrocortisone, insulin, progesterone, and heparin [109]. The vast majority of tumorspheres are cultured based on anchor-free method or cultured on non-adherent surfaces of the plates. In addition, Yang *et al.* also established colon spheroids with hanging drop methodology in several colorectal cancer cell lines [110] and others succeeded in spheroid formation with the rotary cell culture system [111, 112].

**Figure 4:**
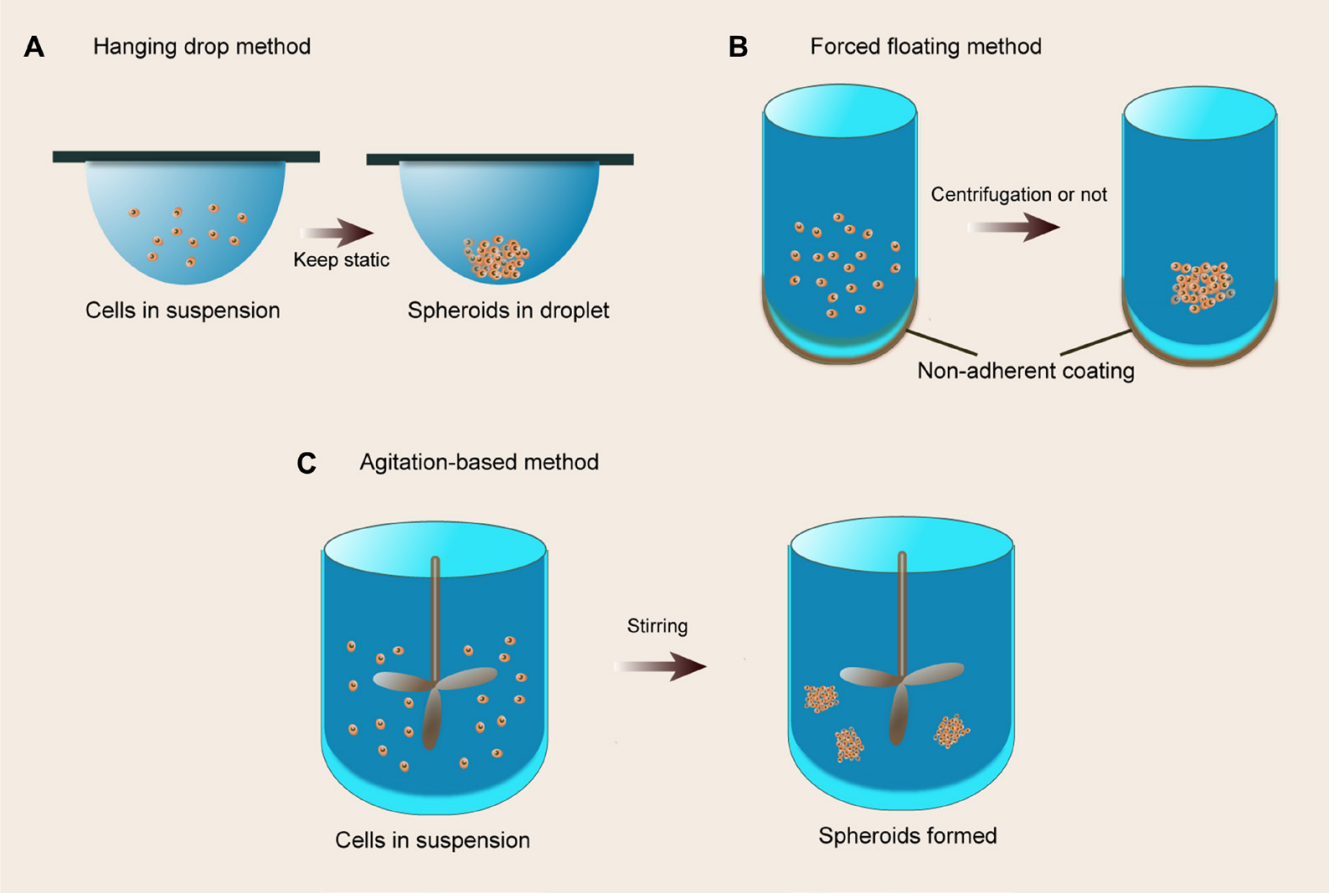
The floating culture methods (**A**) Hanging drop method. A small aliquot (typically 20 ml) of a single cell suspension is pipetted into the wells, and then tray is subsequently inverted and aliquots of cell suspension turn into hanging drops that are kept in place due to surface tensions; (**B**) Forced floating method. A non-adherent coating would prevent cells from attaching to the vessel surface, resulting in forced floating of cells; (**C**) Agitation-based method. A cell suspension is placed into a container and the suspension is kept in motion by gently stirring, resulting in non-adherence to the container walls.

Multicellular spheroids fabricated by floating cells in medium have been developed for many decades. Floating models include hanging drop methods, forced-floating methods (such as agarose- or poly-HEMA-coated plates), and agitation-based approaches (such as spinner flask bioreactors and rotational culture systems), detailedly reviewed in the literature [12, 109]. These systems can be extensively manipulated for emulation of various tissues *in vitro*, including normal tissues, benign and malignant tumors. A schematic of the floating sphere culture systems is presented in Figure 4. Noteworthily, floating spherical models are simplified simulations which only involve cell-cell interactions without cell-matrix interactions although subsequent ECM deposition occurs. Intriguingly, these spheroid culture systems can be integrated with other cell culture systems. For example, floating aggregates can be transplanted on the top of the ECM or entrapped in hydrogels to constitute more complex cell culture models as described below.

The spheroids formed by floating methods can be easily extracted, and recent evidence indicates the value of spheroid cultures in microscale devices for CSCs research [113–115]. Dynamic microwells with circular and square shapes are implemented to control spatial arrangements of multiple cell types in defined geometries. This system can be used to replicate different native biological complexities containing intricate cell-cell interactions and further served as a simplified cancer tissue niche [116]. Microfluidic culture is used to interrogate the cross-talk between melanoma and immune cells and provides conclusive evidence to determine IRF-8 as the key regulator for the interaction [117]. To estimate interaction between cellular microenvironmental cues and CSCs, microfluidic spheroid formation technology is introduced to generate heterogeneous co-culture spheroids. Putative CSCs are cultured inside the co-culture spheroids surrounded by other stromal cells which can be able to maintain the stemness without excessive differentiation [114].

As most of the CSC studies are based on tumorspheres, the lack of heterogeneous cell-cell and cell-matrix interactions might give rise to different conclusions. Implicated by further adoption of spheroids extracted from classic CSC culture, exploitation of more realistic 3D models emulating the *in vivo* situations can provide more credible evidence for CSCs.

### Non-floating sphere culture of CSCs

In the 3D culture system, tumor cells are immersed in a fairly complex microenvironment, which constitutes a compact signaling and functional regulation network with various biological, biochemical, and biophysical factors. 3D cell culture systems can mimic the important nutritional as well as mechanical environments in tumor tissues better than the conventional 2D models. Therefore, the tumor mass formed in 3D culture systems can largely resemble the morphology, histology, and gene expression profiles of the primary cancers [118]. For example, in a 3D culture using Matrigel (a matrix used as 3D support and providing a set of structural and biochemical cues), the HMT-3522 non-malignant breast cells formed organised, polarised acini, similar to those found in healthy breast tissue. However, the HMT-3522 breast cancer cells formed disorganised, loose aggregates. When these cells were treated with antibodies against β1-integrin, the non-malignant cells underwent apoptosis, but the cancerous cells exhibited an apparent reversal back to the normal cell phenotype. A similar result was not observed when the same cells were grown in 2D culture system [119].

#### Cancer tissue-originated spheroids

Cancer tissue-originated spheroids (CTOSs) are generated from tumor fragments via mechanic and enzymatic dissociation of cancer tissue specimens, followed by filtration through cell strainers and finally transplantation on non-adherent culture surfaces [120–122], as illustrated in. CTOS preserves the characteristics of primary tumors. In human urothelial cancer, CTOS retains the differentiation status in the original tumors as revealed by immunostaining [120, 122]. The morphology, protein expression, and vital gene mutations also resemble those of the original tumors. On the other hand, the sensitivity of chemotherapy and radiotherapy with CTOS is in parallel with the therapeutic efficacy *in vivo* in various cancer types [123]. Thus, CTOS can be applied to the detection of the individual sensitivity of cancer therapy and can further facilitate our understanding of resistance mechanisms. Although there is some concern regarding the cell constitution and functional effect of CTOS, several lines of evidence suggest that CTOS contains high level of CSCs and in light of the clear advantages of CTOS, further investigations of CSCs might be implemented with this 3D system.

### Scaffold-based models for CSCs culture

#### Microcapsules

Microcapsules have been extensively investigated in the realm of stem cell and microencapsulated stem cells are verified that remain viability, the ability of potency and directed differentiation [124]. These cell culture systems provide distinct characteristics derived from the materials utilized (such as alginate and agarose), including permeability, mechanical stability, and biocompatibility. It is noted that the concept “microcapsules” referred here is to certain extent overlapped with “spheroids obtained by matrix-like hydrogel encapsulation” which is presented in the later section, as both of these use hydrogels to support the 3D structure. The main discrepancy relies on the inherent feature of microcapsules, i.e., the inert scaffold material, alginate, merely affords physical support for cells rather than provides receptors or signals analogous to the ECM *in vivo*. Thus, this model is also recognized as a “free floating” technique, since only cell-cell and not cell-matrix interaction is involved [12]. Alginate can be cross-linked by various divalent cations, such as calcium and barium, and allows to enclose cells in liquid core [125]. Notably, these inert materials can also be modified with biological and biochemical properties for cell models and be served as matrix resembling technique, as seen below.

**Figure 5:**
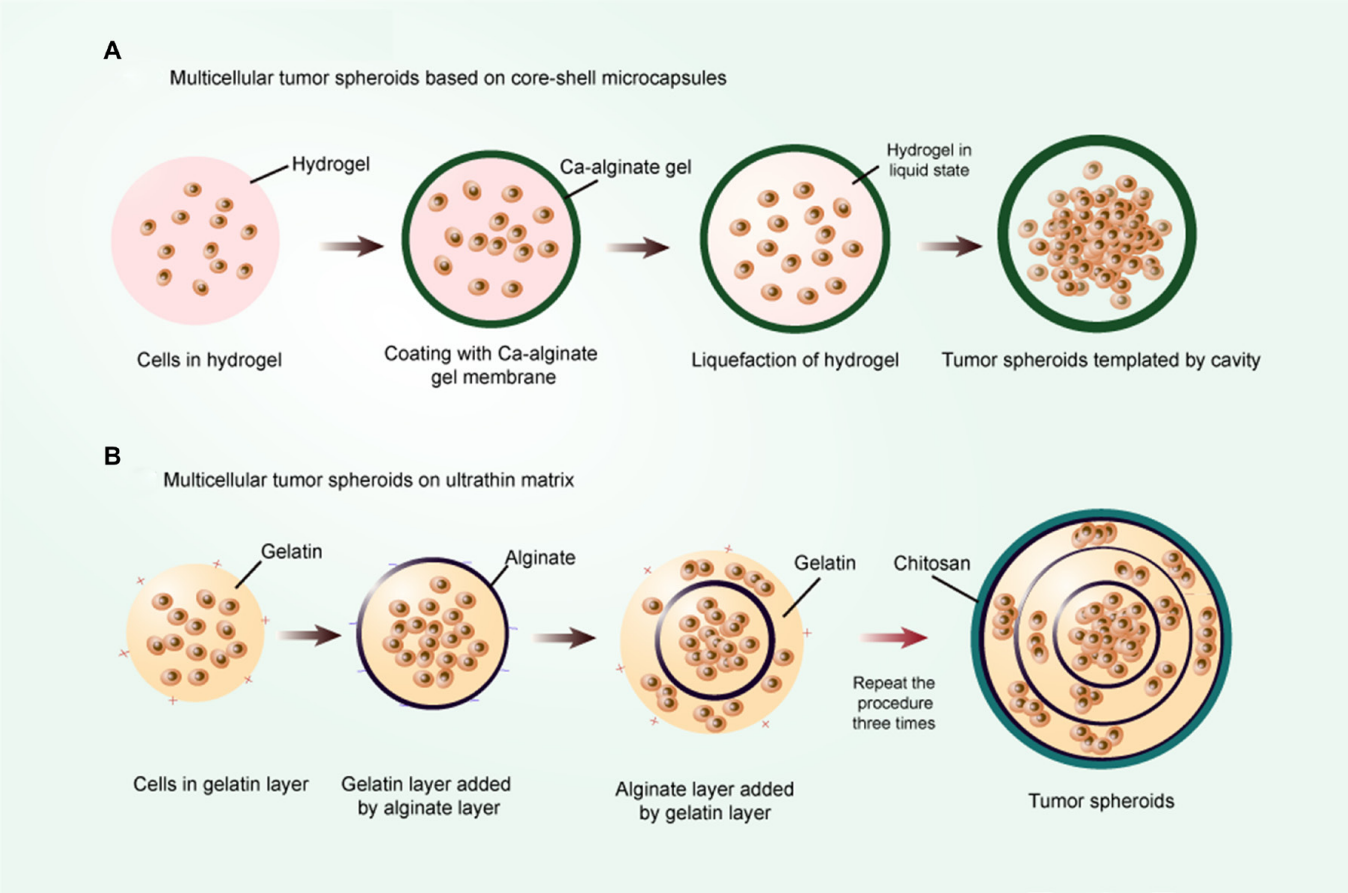
The scaffold-based encapsulation models (**A**) Multicellular tumor spheroids based on core-shell microcapsules. The shell is constituted by an alginate hydrogel of controllable thickness, and the core is constituted by hydrogel of liquefiable property and cells; (**B**) Multicellular tumor spheroid based on ultrathin matrix. First, the gelatin layer is gained by gelatin solution treatment. After that, an anionic polyelectrolyte layer is deposited on the gelatin layer by adding alginate solution. This procedure is repeated three times by the sequential deposition of oppositely charged PE layers. In the end, a cationic PE layer chitosan is deposited on the assembly and the multicellular tumor spheroids are obtained.

Recently, culture of mouse embryonic stem cells in the miniaturized 3D liquid core of core-shell microcapsules is conducted with an alginate hydrogel shell, which maintains stemness better than conventional open-bulk culture [126, 127], as illustrated in Figure 5A. A similar system is applied to maintaining and enriching CSCs *in vitro*, where prostate CSC aggregate is enclosed by alginate hydrogel shell and cultured in the core containing CSC culture medium. Pancreatic CSCs cultivated in porous gels experience thermally-induced liquefaction to form a cavity and could be encapsulated with alginate gel, resulting in the generation of tumorspheres [128]. Furthermore, a mixture of cancer cells and gels in the inner core with the addition of other cell types in the outer layer generates a heterotypic co-culture system [129]. This separate culture model can be used to investigate the paracrine effect between two cell types. Microcapsules exhibit several advantages over floating CSC culture models, including formation of significantly more spheres, shortened culture time, higher expression of stem cell surface makers, and greater tumorigenicity.

#### Spheroids obtained by matrix-like hydrogel encapsulation

Apart from the 3D culture systems depicted above, the spheroids can also be originated from matrix-like hydrogel encapsulation, such as hyaluronic acid, matrigel, and collagen. These polymer compositions and derivatives are capable of creating 3D scaffolds and recreating native ECM-like environment *in vitro* [130, 131]. Actually, these mono-compositions of scaffolds can only represent one component of the complex ECM network in formation and function. To fabricate this 3D model, several approaches and constructs have been exploited. First and most common is direct mixture of matrix-like hydrogel with cells in suspension. For example, the hyaluronic acid (HA) derivative HAALD, which exhibits reduced solution viscosity and allows cells to be readily dispersed without any noticeable cell damage, upon addition of another HA derivative with enhanced solution viscosity, exhibits stable sphere formation [132]. Second, cell aggregations are initially produced by growing cell suspensions in low-attachment plates, and the free-floating spheroids are then transferred to certain gels amenable to generating well-appearance cell models [133]. Another technology utilizes layer-by-layer ultrathin film to form a niche-like matrix, which then assembles to generate a multicellular tumor spheroid microenvironment [134], as illustrated in Figure 5B.

Materials of fabricating scaffold-based 3D models can be further modified in structure and function, resulting in controllable and reproducible variations to investigate the effect of matrix cues on cell biology. Peptides such as arginine-glycine-aspartate (RGD) integrin-binding motif and various growth factors can be conferred on the matrices, such as polyethylene glycol (PEG), through factor XIII-catalysed cross-linking [135, 136]. To investigate the sensitivity of certain matrices to degradation by cell-secreted/activated proteases such as matrix metalloproteinases (MMPs), MMP substrates can be precisely attached to the hydrogel network [137, 138]. In addition, TGF-β1 can be loaded into gelatin microparticles (MPs) and peripherally encapsulated with oligo(poly(ethylene glycol)) fumarate (OPF). By altering the OPF formulation and the cross-linking extent of gelatin MPs, release of TGF-β1 can be easily controlled [139].

One characteristic of the matrix-like scaffold model is that stem cells can be further induced to give rise to desirable lineage differentiation. The addition of RGD-integrin binding in the cell-ECM confers an enhanced chondrogenic commitment and cartilaginous tissue formation on mesenchymal-like cells from embryoid bodies (EBs) when cultured in a 3D PEG-based hydrogel matrix [136]. Analysis of the global gene expression profile of embryonic stem cells cultured in Cytomatrix™ scaffolds indicates that these cells express significantly higher levels of key genes that increase ECM production, growth factor and cytokine activity, as well as cell growth and differentiation [140].

#### Organoids

Organoid models are generated from single cells or from pre-aggregated pluripotent stem cells, progressing to complex and sophisticated structures by division and expansion of stem cells *in vitro*. Initially, this tissue engineering approach is designed to generate a 3D construct with some distinct characteristics of the intestine, including establishment of crypt-villi architecture and lumenized interior from intestinal crypt fragment which exclusively contains Lgr5^+^ stem cells [141, 142]. Evoked by the pioneering work in intestinal organoids, in recent years many diverse organoids are reported, including cerebral, liver, pituitary gland, inner ear, pancreas, and hair follicle [143–145]. Technologically, the culture system is constituted by Matrigel and cell medium containing various factors which differ within diverse tissues [142, 146]. For example, factors including R-Spondin, EGF, and Noggin are essential for generating intestinal organoids. A liver bud is generated with aggregation of three cell types (human umbilical vein endothelial cells, human MSCs, and iPSC-derived hepatic cells) at very high cell densities [147]. The primary organoids generated from stem cells can be dissociated into single cells to form new organoids, but at a rather low efficiency. The organoids derived from either single stem cells or whole crypts are indistinguishable in appearance. In both situations, the stem cells are located at the bottom of the crypt, and the fully polarized enterocytes line up in the central lumen [141].

Albeit substantial observations have confirmed that stem cells preserve the ability of generating a rough emulation of organs in appropriate conditions, whether this is also true for cultured cells and model tumors is not known. Intestinal adenoma can be generated from Lgr5^+^ stem cells in mice and isolated intestinal adenomas can form cystic organoid structure without budding *in vitro* [148]. Consistent with normal tissue organoids which contain various differentiated cell types, such as neuroendocrine cells, goblet cells, and enterocytes in intestinal organoids, adenoma cultures also exhibit significant heterogenesis. The existence of heterogeneous cells in adenoma cultures indicates that differentiation towards distinct epithelial lineages might occur at all stages of tumors progression [149]. Generally, the niche cues pivotal for CSC differentiation and organization are less characterized and organoids generating from CSCs have not been investigated.

#### Heterotypic 3D co-culture models in ECM

**Figure 6:**
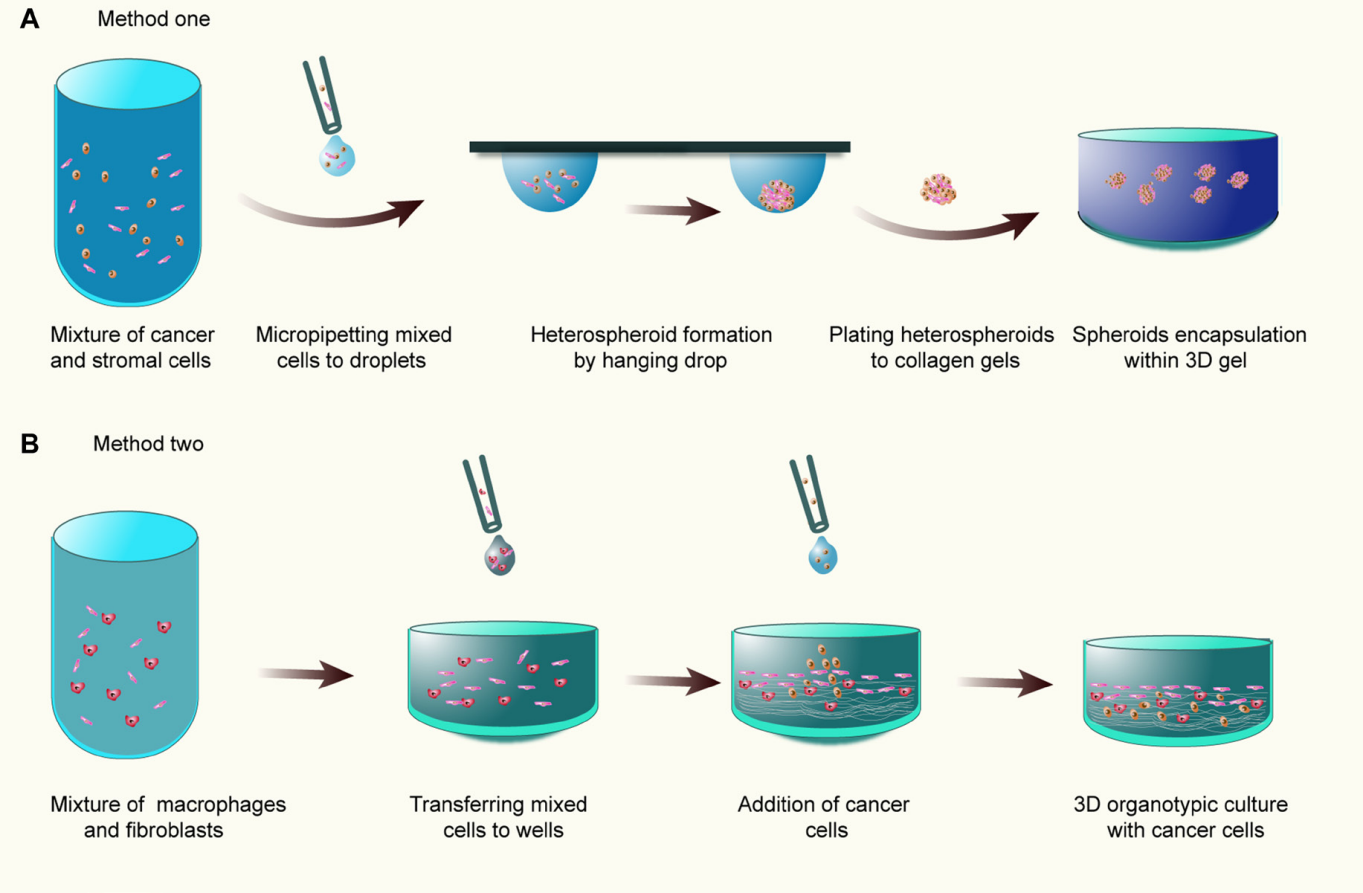
The organotypic culture models (**A**) Tumor cells and stromal cells are mixed to form heterotypic MCTS, and then transferred to and encapsulated in 3D collagen gel; (**B**) Fibroblasts and macrophages are directly mingled with collagen gel and poured into wells to polymerize, followed by tumor cell placement on the top of wells.

Organotypic culture (OTC) models or heterotypic 3D co-culture models in ECM have attracted enormous attention for the investigation of the histological, physiological, and functional interactions between cancer cells and stromal cells. OTCs are simplified emulation of tumor tissues which simultaneously contain various elements within tumors, including heterotypic cell-to-cell interactions, cells residing in ECM as well as nutrient and gas gradients. Initially, this model is envisioned to establish skin equivalents or dermal equivalents *in vitro* comprising ECM-like gels and incorporated fibroblasts [150]. Evoked by these implements, organotypic tumor co-cultures based on dermal equivalents are adopted to investigate the effect of stromal cells or niche factors such as cytokines on tumor development [151, 152]. Currently, several approaches for constructing organotypic tumor models are developed. First, tumor cells and stromal cells are mixed to form heterotypic multicellular tumor spheroids MCTS, and then encapsulated in non-polymerized ECM gels, and followed by gelling [12, 153, 154] (Figure 6A). Second, analogous to classical dermal equivalent models, fibroblasts and macrophages are directly mingled with collagen solution and poured into wells to polymerize, followed by tumor cell placement on the top of wells [151, 152, 155, 156] (Figure 6B).

OTCs exhibit several advantages for cancer research. PDVA tumor cells develop a multilayered tumor epithelium on the top of dermal equivalents, and the areas of tumor cells infiltrating collagen-I dermal equivalent are only detectable in OTCs containing macrophages or macrophages together with fibroblasts [151]. In a systemic study with colon cancer OTCs, this model mimics the cellular architecture of human cancer tissues in histology and phenotype, and also emulates activation of similar signaling pathways and gene expression profile [153]. Intriguingly, a normalization of the epithelial structures is found at early culture period, while invasive potential of colon cancer cells can be induced in this co-culture system as well. In CSCs, niche plays a vital role in the pathophysiology of CSCs. While the heterogeneous co-culture systems are mostly presented by the oversimplified co-culture systems, the effect of stromal cells on CSCs might not be reflected on such models. Thus, application of OTCs to CSC research is a feasible solution to address the missing link between the conditions *in vivo* and the oversimplified 3D models *in vitro*.

### Microfluidic devices for CSC culture

Microfluidic technology attracts great enthusiasm and practices in cell cultures, micro-tissues and micro-organs fabrication *in vitro*. Although the majority of microfluidic channel-based systems only support 2D cultures, progress has successfully added a third dimension to the application of this biological model in recent years [157]. Faithfully, microfluidic systems are able to recapitulate the properties of tissues, such as spatial crosstalk with microenvironment in 3D, vascularization, perfusion, and gradient formation for nutrients and oxygen [158, 159]. The culture medium is precisely controllable with perfusion instead of static condition, and interstitial flow can be imitated with tunable chemical and biomolecular gradients by spatiotemporal administration [12]. Microfluidic culture system with high compatibility also allows medium recirculation, which facilitates examination of cell metabolism and drug toxicity [160]. In addition, microfluidic devices can be used to culture diverse cells in separate micro-chambers and connect each of them via narrow capillary migration micro-channels to build up effective interactions and provide conclusive evidence for paracrine factors [117]. The development of microengineering in regeneration research has expedited microfluidic applications from cells-on-chips to organs-on-chips [161–163]. Thus microfluidic devices can endow dynamic medium exchange mimicking *in vivo* environment and provide multiple levels of complexity in tissue simulation.

To integrate 3D tissues into micro-chambers, two primary methods can be utilized. Cell aggregates or spheroids are pre-produced in dedicated off-chip systems and then transferred into microfluidic chip and positioned with gravity [164, 165]. To prevent the spheroid from adhering to microchannels or generating bubbles, the coating material which renders the surface hydrophilic is introduced prior to import of spheroid [165]. Off-chip spheroid fabrication allows decoupling from in-chip culture, so that the complexity of tissue fabrication does not impact the layout of the microfluidic chip. On the other hand, in-chip spheroid formation is also implemented for high-throughput screening of drugs. The process of spheroid formation includes generation of hydrogel droplets containing cells in suspension, and *in situ* gel droplets in droplet incubation array [166]. When hydrogel solution is mixed with two or more cell types, heterotypic culture will be established. These systems allow a wide range of *in situ* bioanalysis, such as microscopic observation of cellular morphology, fluorescent staining as well as fluorimetric measurements. Moreover, new emergence of automatic and digital microfluidics provides a powerful tool for spheroid-based assays [115].

The cells-on-chip system is designed to mimic the migratory behavior of brain tumor stem cells in a space-compartmentalized microfluidic device [167]. This system creates an active microenvironment for regulating the migration of brain tumor stem cells and reflecting diverse stages of tumor with successive cellular morphology transformation. A simplified microfluidic system is designed and performed to screen aptamer targeting agents with high affinity for colorectal cancer cells and colorectal cancer stem cells [168]. Another study presents a cost-effective and purpose-tailored 3D spheroid culture platform to identify EMT/MET process, as well as effectively enrich CSCs [169].

Considering that most spheroids are generally not clonal and do not exclude the possibility of cellular aggregation, a scalable single-cell suspension culture chip can provide single-cell isolation, tracking, and continuous medium perfusion to enrich CSCs clonally [170]. To investigate tumor progression, a biomimetic microengineering approach is developed based on 3D microsystem principles [171]. Microfluidic co-culture of multicellular ductal carcinoma *in situ* (DCIS) spheroids is embedded into 3D ECM scaffolds. The invasive ductal carcinoma can be mimicked by this integrated system. Additionally, microfluidic devices can be applied to investigate, predict, and analyze the process of stem cell differentiation. In a microfluidic array, neural stem cells in 3D collagen hydrogel are introduced into the central microchannels and co-cultured with MSCs in two side channels. The presence of MSCs clearly induces enhanced differentiation of neural stem cells into neuronal lineage [172]. Similar study indicates that aggregates of neurospheres are readily induced to form neurons in microfluidic culture system with high efficiency [173].

### Metastatic models and CSCs

Metastasis is limited to *in vivo* mouse models and *in vitro* models in which tumor cells are cultured in matrices under various mechanical and chemical cues. While the disadvantages of *in vivo* models are obvious, including difficulty to perform tightly regulated parametric studies and quantification [158], the widely accepted Boyden chamber and transwell assays are less controllable over local environment and cellular interactions. Recent investigations lead to new *in vitro* methodologies utilizing the emergent technologies of microfluidics combined with 3D culture systems, detailedly reviewed in the literature [158, 174]. The initial event of metastatic cascade, intravasation, can be simulated by microfluidic device. Metastatic breast cancer cells with controlled interstitial flow in a 3D micro-chamber exhibit migratory behavior and migrational speed can be further determined [175]. Breast cancer cells generate protrusions and migrate along with the gradient of growth factors such as EGF within 3D basement membrane gels [176]. Additionally, microfluidic devices are designed to recreate the process of extravasation via adherence to the endothelial monolayer under physiological flow conditions [177]. Taken together, 3D metastasis models can be employed to study metastatic cascade.

Circular chemorepellent-induced defect is highly reminiscent of the defect displayed in the lymphovascular walls at tumor invasive sites *in vivo* [178]. Circular chemorepellent-induced defect assay can excellently mimic the initial process of metastasis *in vitro*. The 3D configuration can recapitulate the aggressive tumor and lymphatic endothelial invasive barrier constituted by lymphendothelial cells [179]. Interestingly, tumor aggregate exhibits enhanced expression of CD44, ICAM1, and VEGFA, which are over-expressed in metastatic cancers. The underlying mechanisms that tumor cells invade the lymphatic system include over-expression of the arachidonate lipoxygenase ALOX15 and activation of the NF-κB signaling pathway [180]. Recently, novel organotypic corticostriatal rat brain slice culture is used for implantation of spheroids as an alternative to artificial gels. Intriguingly, all primary spheroids are enriched for putative CSCs markers, including Sox2, Bmi-1, and nestin, and these characteristics are preserved in organotypic spheroids [181]. Invasion-competent subpopulation is demonstrated with mesenchymal traits, such as reduced expression of E-cadherin and increased expression of vimentin [182]. Notably, collective invasion and aggressive cells can enhance the migratory ability of non-invasive epithelial cells [182, 183]. Another study with a 3D micro-organoid tumor model indicates that co-culture of spheroids with adjacent fibroblasts induces robust invasion into the ECM-like gel [184]. In this process, tumor cells are transformed to the epithelial phenotype on account of the tumor-stroma interaction induced by TGF-β and PDGF signaling. Taken together, 3D metastatic models can potentially reflect the CSCs in metastasis.

## PERSPECTIVES

Accumulating evidence supports that niche plays an essential role in the maintanece of CSC behaviors and functions. Understanding the complex interactions between CSCs and niche should help provide useful information about: 1) the origin of the CSCs of different states; 2) the signaling mechanisms underlying the CSC biology; and 3) the treatment options targeting CSCs. 3D culture models offer the advantages of simulation of the interactions between CSCs and niche and recapitulation of the spatial dimension of tumor microenvironment. Inspired by the advances in tissue regeneration and stem cell biology, we speculate that CSCs in optimal culture conditions can intimately resemble the morphology, phenotype, and heterogeneity of tumor tissues. To achieve this, several essential issues should be considered. First, introduction of dynamic perfusion to emulate the blood flow might surmont the hindrance in conventional 3D models which merely simulate the avascular microenvironment. Second, microscale devices allow long-term culture, controllable interstitial flows, defined shapes and positions that can be used to position cells and tissues, as well as highly structured 3D culture microenvironment [157, 162]. Third, tissue engineering based on matrix-like scaffold, such as 3D printing technology, can be used to improve the sophisticated fabrication of tumor tissues *in vitro*. Combination of tissue engineering and microscale devices would open a new avenue to the development of more sophisticated 3D tumor models that suit the increasing needs of CSC research. Notablly, the 3D models of organoids deserve more attention due to the high level of simulation of tumor tissues and biocompatibility when introduced into animals. In addition, worthy of investigation are the techniques that grow CSCs directly from diverse primary tumor tissues and the 3D models that deciper the complex mechanisms of therapy resistance of CSCs.

## Abbreviations

3D: Three-dimensional
CAF: Cancer-associated fibroblast
CCID: Circular chemorepellent-induced defect
CSC: Cancer stem cell
CTC: Circulating tumor cell
CTOS: Cancer tissue-originated spheroid
ECM: Extracellular matrix
EMT: Epithelial-to-mesenchymal transition
iPSC: Induced pluripotent stem cell
MCTS: Multicellular tumor spheroid
MET: Mesenchymal-to-epithelial transition
MSC: Mesenchymal stem cell
OTC: Organotypic culture
PDGF: Platelet-derived growth factor
SP: Side population
TAM: Tumor-associated macrophage
VEGF: Vascular endothelial growth factor
